# Stereotactic ablative body radiotherapy (SABR) for inoperable, chemorefractory retroperitoneal lymph node relapse from non seminomatous germ cell tumour of testis: a case report

**DOI:** 10.1259/bjrcr.20160114

**Published:** 2018-03-14

**Authors:** Abhishek Gulia, Anil Kumar Anand, Anirudh Urumi Punnakal, Amit Kumar, Ch Kartikeswar Patro, Anil Kumar Bansal

**Affiliations:** 1 Department of Radiation Oncology, Max Super Speciality Hospital, Saket, India; 2 Department of Radio-diagnosis, Max Super Speciality Hospital, Saket, India; 3 Division of Medical Physics, Max Super Speciality Hospital, Saket, India

## Abstract

We describe a case of non-seminomatous germ cell tumour (NSGCT) of the testis with oligorecurrence in para-aortic nodal mass, which was inoperable and chemorefractory. Conventionally fractionated radiotherapy in this setting is generally believed to achieve poor results, because the dose is limited by the tolerance of surrounding normal tissues. Use of stereotactic ablative body radiotherapy (SABR) for para-aortic nodal recurrence from a few sites has been reported; its application in NSGCT has not been described in literature to our knowledge. SABR allowed us to deliver highly precise, ablative dose of radiation to the recurrent para-aortic nodal mass with long-term disease control (more than 6 years). The ablative dose delivered with SABR proved to be effective in NSGCT, traditionally considered radioresistant. While, in the present case SABR was delivered due to the inoperability of the lesion, further data on its successful use in NSGCT recurrences is warranted.

## Background

The oligorecurrent state was defined by Niibe et al as the state in which the patient shows distant relapse in only a limited number of regions while the primary site of cancer is controlled, allowing a curative therapeutic strategy.^1^ Local therapy such as surgery and radiotherapy for the relapsed site(s) could thus improve the patient’s survival.^1^

In non-seminomatous germ cell tumours (NSGCTs), para-aortic failure after chemotherapy and retroperitoneal lymph node dissection (RPLND) is reported in approximately 12% of patients.^2^

Conventionally fractionated radiotherapy in this setting is generally believed to achieve poor results, because the dose is limited by normal tissue tolerances of the surrounding organs at risk such as bowel, kidney, liver and spinal cord. Local control rates with conventional radiotherapy in patients of carcinoma cervix with isolated para-aortic recurrence have been reported in the range of 33 to 50%.^3, 4^

Stereotactic ablative body radiotherapy (SABR), a form of high-precision radiotherapy, is characterized by the delivery of high biological doses of radiation to a well-defined target in 3–5 fractions. In the setting of oligorecurrent disease confined to para-aortic lymph nodes (PALN), SABR has been used in patients with carcinoma cervix, endometrium, prostate and stomach, with local control rates of 70–80%.^4, 5^

However, there is no published data to our knowledge on the use of SABR in PALN oligorecurrence in a case of non-seminomatous germ cell tumour.

## Clinical Presentation

A 38-year-old male presented in 1994 with left testicular swelling and a conglomerate of enlarged PALN around the left renal hilum (measuring 6 × 4 cm) evident on contrast-enhanced CT scan (CECT) of the abdomen. He was diagnosed to have NSGCT Stage IIB and underwent high inguinal orchidectomy on 6 December1994 at an outside hospital. Histopathological report showed NSGCT—endodermal sinus subtype. Serum alfa fetoprotein (AFP) (380 ng ml^−1^) and beta human chorionic gonadotropin (hCG) (379 mIU ml^−1^) were raised. Thereafter, he received four cycles of chemotherapy with bleomycin, etoposide and cisplatin (BEP) regimen from December 1994 to April 1995.

Post chemotherapy, in May 1995, the AFP and beta hCG returned to normal range and CT abdomen showed a residual node (2.2 × 1.6 cm) with central necrotic area at the level of L1−L2 vertebra. The patient was kept on regular follow-up with regular monitoring of serum AFP and beta hCG levels, which remained within the normal range till October 1995.

In November 1995, CT abdomen revealed an increase in size of the retroperitoneal lymph node mass to 5.2 × 4.1 × 3.4 cm. However, the serum tumour markers were still in the normal range. In January 1996, RPLND was performed and histopathology was reported as necrotic lymph nodes with reactive lymphoid hyperplasia. The patient remained asymptomatic and disease free with normal serum markers for 8 years, till June 2003.

In July 2003, serum AFP level rose to 600 ng ml^−1^ while the beta hCG was still undetectable and serum lactate dehydrogenase level was 396 IU l^−1^. CT scan abdomen showed relapse in PALN (size 6.5 × 5 cm) abutting the left renal vessels and left renal pelvis. The patient again underwent exploratory laparotomy with excision of retroperitoneal mass. Histopathology revealed mixed germ cell tumour, comprising predominantly the yolk sac variety with focal areas of embryonal carcinoma. Subsequently, he received three cycles of chemotherapy with BEP regimen till November 2003.

For the next 5 years, he again remained disease free with normal serum marker levels and no abnormal findings on CT scans.

In February 2008, routine CT abdomen and chest showed 1–1.5 cm sized lymph node in the left para-aortic region at the level of the left renal hilum. Ultrasound-guided fine needle aspiration cytology (FNAC) of the lymph node was done, which revealed recurrence of the germ cell tumour. He was again given six cycles of chemotherapy with cisplatin and etoposide and remained well till August 2009 (Table 1).

**Table 1.** Serial values of size of retroperitoneal lymph node mass and corresponding SUVmax and AFP values

Date of PET-CT	Size of retroperitoneal LN (cm)^*a*^	*﻿*SUVmax	AFP (ng ml^−1^)
25.08.2008	1	1.3	6.96
08.12.2008	1.2	2.9	6.43
19.05.2009	1.75	8.1	9.26
11.08.2009	1.9	9.4	20.11
22.02.2010	3.5	9.9	45
SABR delivered on 11.03.2010			
11.08.2010	2.5	4.5	5.26
05.02.2011	2.0	2.3	4.56
27.06.2017	CT scan not done	PET not done	4.35

AFP, alpha fetoprotein; FDG, fludeoxyglucose; LN, lymph node; SUV, standardized uptake value.

a The maximum diameter in the short axis of axial CT slices.

## Treatment

**Figure 1.**
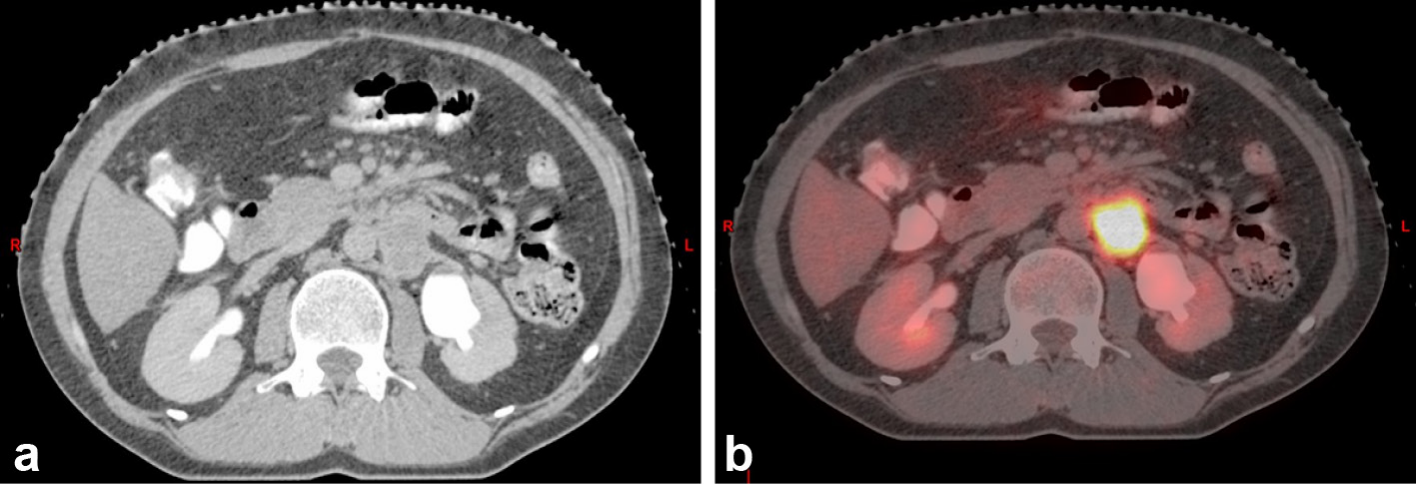
Pre-stereotactic body radiation therapy contrast-enhanced CT abdomen (a) and CT-positron emission tomography scan (b) show fludeoxyglucose-avid retroperitoneal lymph node mass 3.5 × 3.2 cm in size with dilated left renal pelvis.

The patient came to us in February 2010 in view of rising markers and increase in size of the retroperitoneal nodal m ass adjacent to the left renal hilum. The mass was 3.5 × 3.2 cm in size and was adherent to the aorta and left renal vessels (Figure 1). Left renal pelvis dilation was also observed probably due to post operative periureteric fibrosis after two previous surgeries. A whole body CT-positron emission tomography (CT-PET) revealed no other metastases. His case was discussed in the tumour board and the nodal mass was deemed inoperable. An option of SABR was offered to the patient due to lack of other effective alternatives in this inoperable and chemorefractory recurrence. Also, PALN recurrence had occurred at the same site despite two surgeries and chemotherapy, over a period of 15 years.

**Figure 2.**
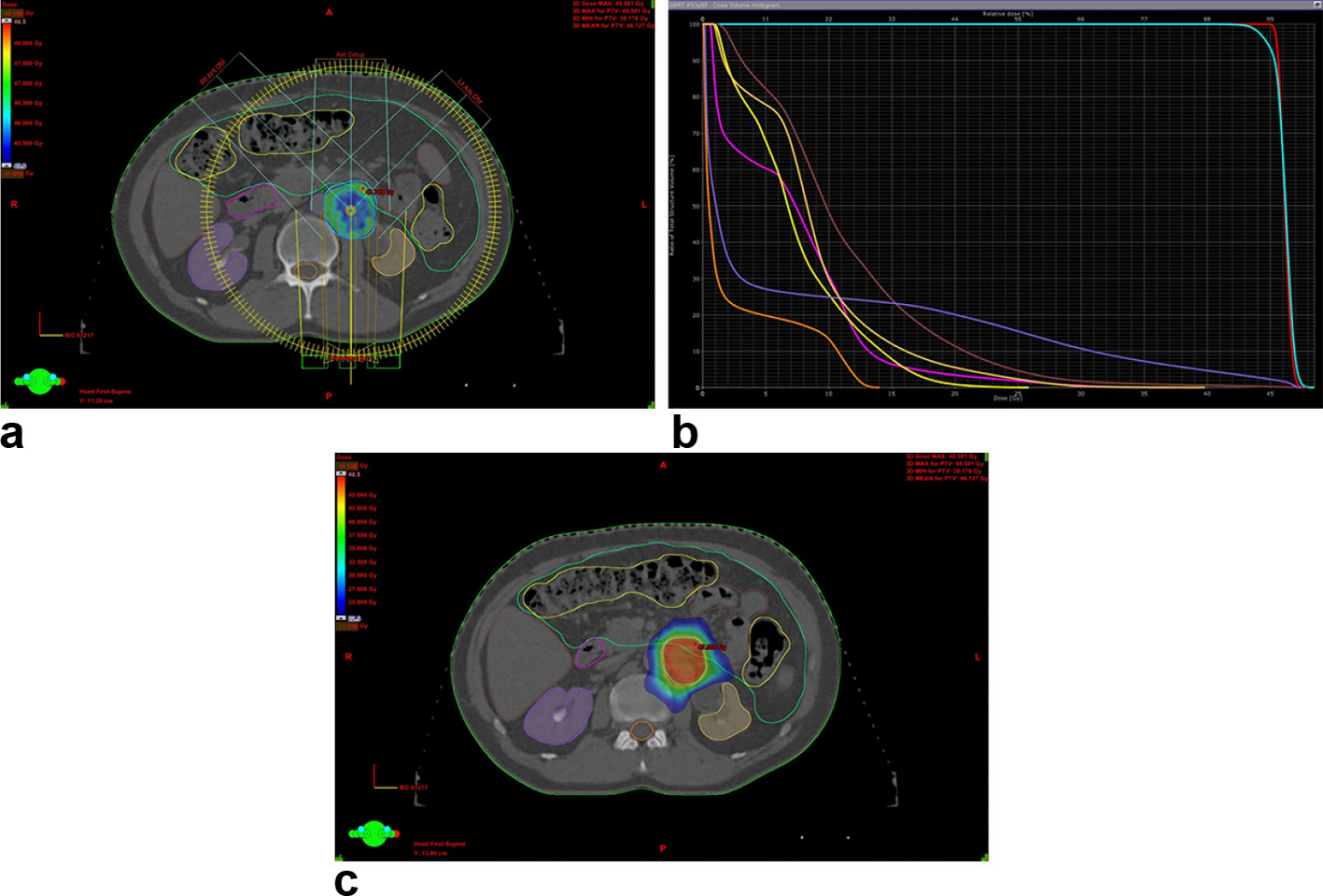
Dose distribution in a range from Dmax 48.5 Gy to the prescribed dose 45 Gy, achieved with SABR by volumetric modulated arc therapy (a), DVH parameters (b) and low-dose spill in surrounding organs at risk (c), with dose range from Dmax to 50% of the prescribed dose. Red–GTV, cyan–PTV, brown–small bowel, mustard–left kidney, purple–right kidney, yellow–large bowel, green–bowel bag, magenta–duodenum, violet–vessel large (aorta) and orange–spinal cord. DVH, dose volume histogramPTV, planning target volume.

The patient was immobilized with six clamp thermoplastic cast (Orfit, Wijnegem, Belgium) and a planning CT scan with 2 mm slice thickness. The target volume included gross nodal disease (GTV) apparent on CT-PET scan. Planning target volume (PTV) was 3 mm in the axial and 5 mm in the craniocaudal direction. In view of the location of the tumour in close proximity to the aorta, left kidney and the surrounding small intestine, a fractionated dose of 45 Gy in 6 fractions was chosen. This regimen has a BED10 (biologically effective dose) value of 79 Gy, which was desirable in view of the radio-resistant histology. A dose of 45 Gy/6 fractions was prescribed to the PTV and was delivered by volumetric modulated arc therapy (VMAT) technique while sparing the organs at risk (OAR) (Figure 2). As per dose volume histogram (DVH) analysis, 95% of the PTV received 44.8 Gy, with normalization to 99% isodose line (GTV D90% 45.6 Gy, D95% 45.4 Gy and PTV D90% 45.3 Gy, D95% 44.8 Gy). The final treatment plan had a conformity index of 0.94 and a geometric index of 3.1. The OAR dose limits suggested in American Association of Physicists in Medicine Task Group 101 report were followed for SABR plan evaluation.^6^ The dose constraints for great vessels (aorta) (D10 cc should not be more than 47 Gy and Dmax should be less than 53 Gy) in 5 fractions was prescribed.^6^ Doses to other surrounding OAR are depicted in Table 2. Image guidance was done daily with ExacTrac X-ray 6D system (BrainLAB AG, Feldkirchen, Germany). On the first day, image guidance was done with both ExacTrac system and cone beam CT scan.

**Table 2.** Dose volume parameters achieved with SABR

	Threshold volume (cc)	Threshold dose (Gy)	Maximum point dose (Gy)
Vessel Large (Aorta)	10	18	47.2
Duodenum	5	14.1	33.8
10	12	
Liver	700	0.2	
Bilateral kidneys	200	0.3	
Spinal cord	0.35	12.9	13.5
1.2	12.3	
Bowel large	20	15.6	25.1
Bowel small	5	26.6	47.5

Currently, there is no proven role of prophylactic radiation to para-aortic nodes after RPLND and chemotherapy even in multiple involved lymph nodes in NSGCT. It seems to be due to limitation of delivery of high dose of radiation to the whole para-aortic region and the radio-resistant histology, which needs high dose of radiation for durable local control. That is why we opted for SABR, which can deliver a high dose to a precise volume while sparing the surrounding critical normal structures such as duodenum, small bowel, spinal cord and vessels.

**Figure 3.**
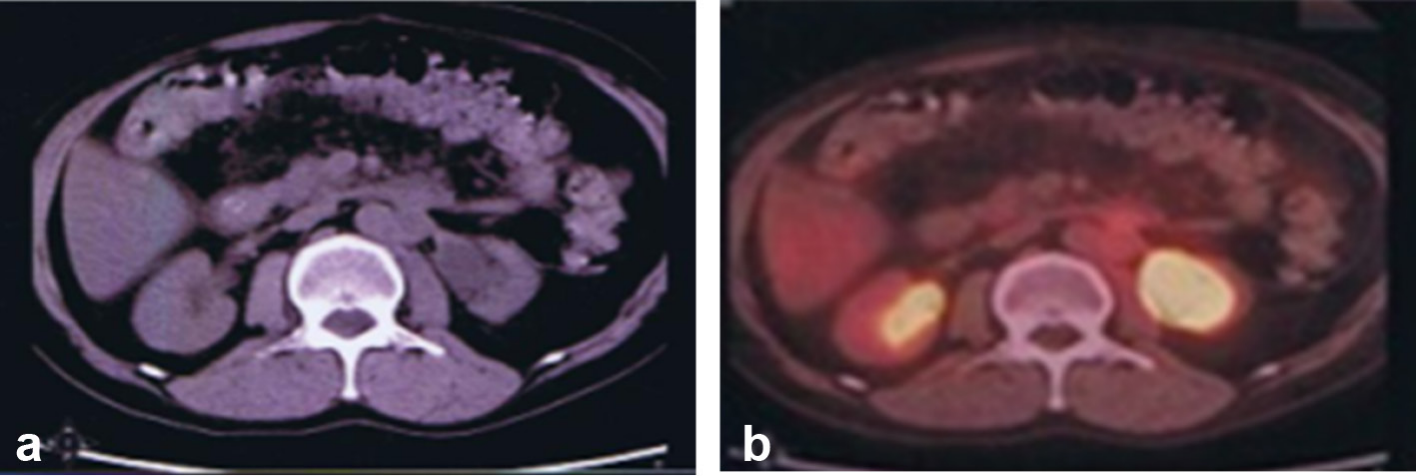
Contrast-enhanced CT abdomen (a) and CT-positron emission tomography scan (b) 11 months post stereotactic ablative body radiotherapy shows marked reduction in left para-aortic mass with no significant FDG uptake, while the dilated left renal pelvis persists.

The patient tolerated the treatment well and had only Grade II gastrointestinal toxicity (RTOG Acute Radiation Morbidity Scoring Criteria) in the form of on and off abdominal pain, which resolved with analgesics. Post SABR, CT-PET done in August 2010 (8 months later) revealed regression of the lymph node mass with faint FDG avidity and central necrosis (Figure 3). Serum AFP declined to 4.08 ng ml^−1^ from the pre treatment value of 45 ng ml^−1^.

## Follow-Up

Since then the patient has been on 3 monthly follow-up with serum markers and PET-CT scan in the first year and then CECT abdomen at 4–6 months interval. Currently, the patient is disease free 6 years later, with normal serum markers and normal CT scan of the abdomen. He has no long-term adverse effects.

## Discussion

Use of SABR to treat oligometastases has been reported in quite a few studies. Lung, liver, brain and bones remain the most commonly treated sites, and colorectal, breast, prostate and lung are the most common primaries.^7^ Results are encouraging in the treatment of lung and liver metastases but less clear in the treatment of abdominal lymph nodes and adrenal gland.^8^ Use of SABR to treat para-aortic nodal oligorecurrence in NSGCT has not been reported yet to our knowledge.

The aim of SABR is to achieve durable local control at the oligometastatic site, which can translate into longer survival in some patients. SABR in this setting is advisable in highly selected group of patients with controlled primary, preferably less than five sites of metastases, oligometastasis <5 cm in size, young age and good performance status.^1^ Our patient fulfilled all these criteria.

Conventional radiotherapy for PALN oligorecurrence has been studied in the past. Niibe et al treated 84 patients of carcinoma cervix with conventional radiotherapy (mean dose 50.8 Gy, 2 Gy per fraction) achieving a 5-year OS of 31.3% with no patients having Grade 3 or greater late toxicity.^9^ Kim et al^10^ reported 12 patients of carcinoma cervix treated with 60 Gy hyperfractionated radiotherapy with 2 daily fractions of 1.2 Gy each, achieving a complete clinical tumour response in 33% (4/12) and partial response in 67% (8/12) at 1 month post treatment with a 3-year OS of 19%. Grade 3–4 haematological toxicity was seen in 2 patients. Six (50%) patients experienced Grade 2 nausea. Subsequent distant metastases after PALN treatment developed in seven (58%) patients.^10^

With recent advances in the areas of immobilization, motion management, planning techniques and on-board imaging, it has become possible to treat well-defined target volumes with high ablative doses, while saving the surrounding OARs. Choi et al treated isolated PALN recurrence from carcinoma cervix or endometrium by stereotactic body radiation therapy (SBRT) and achieved a 4-year OS of 50.1% and a 4-year actuarial local control rate of 67.4%.^11^ The largest series of treatment of unresectable lymph node metastases in retroperitoneal region has been reported by Bignardi et al.^12^ The authors have reported outcome in 19 patients with unresectable nodal metastases in retroperitoneal region treated with SABR. A dose of 45 Gy in 6 fractions was delivered with 3-dimensional conformal radiotherapy (3DCRT) in 11 patients and with VMAT in 8 patients. The actuarial rate of freedom from local progression was 77 ± 13.9% with both techniques, at 12 and 24 months. The number of metastases (solitary versus nonsolitary oligometastases) emerged as the only significant variable affecting progression-free survival PFS (*p* < 0.0004). Both acute and chronic toxicities were minimal in this study. The authors concluded that SBRT for retroperitoneal lymph nodes was feasible with good clinical results.^12^

The proposed mechanism for oligorecurrence by Niibe et al states that at the time of treatment for the primary lesion, the oligorecurrent patient might have one to clinically undetectable micrometastases, which remain dormant for a period and then grow and become clinically detectable by CT, MRI, PET or increasing tumour markers.^1^ The patient referred in this case report has achieved long-term survival (possibly a cure) with local therapy alone while there was always a possibility of other micrometastatic sites becoming clinically detectable by now as per the known natural history of the disease. It is postulated that radiation therapy (RT), in addition to the direct effects on tumours, may also contribute by making tumours visible to immune system.^13^ The ensuing immune response promotes the expression of inflammatory and immunostimulatory mediators, which act on neighbouring, non-irradiated cells leading to “bystander effect.” Systemic effects can also occur in non-irradiated areas (out of field) after treatment with localized radiation. These effects are called “abscopal effects” and appear to be immune mediated, particularly by adaptive immunity.^13^ The immune mediated effect is more pronounced with high dose per fraction and this effect might also contribute towards better survival outcomes for SABR patients than conventional radiotherapy.^14^

High-dose RT delivered with SABR in the present case study has yielded complete response in oligometastases in retroperitoneal lymph node. It has translated into a long-term survival for this patient.

## Learning points

1. SABR is technically feasible for oligometastases in retroperitoneal nodes. It allows delivery of ablative radiation dose to the tumour sparing the surrounding OAR’s tolerance limits.
2. SABR with high dose can result in durable local control, which has a potential to provide long-term survival even in radioresistant tumours such as NSGCT.
3. SABR to para-aortic lymph node mass was associated with minimal toxicity and was well tolerated.

## References

[1] Niibe Y, Hayakawa K Oligometastases and oligo-recurrence: the new era of cancer therapy. Jpn J Clin Oncol 2010; 40: 107–11. doi: 10.1093/jjco/hyp167 20047860 PMC2813545

[2] Heidenreich A, Thüer D, Polyakov S Postchemotherapy retroperitoneal lymph node dissection in advanced germ cell tumours of the testis. Eur Urol 2008; 53: 260–74. doi: 10.1016/j.eururo.2007.10.033 18045770

[3] Macdermed DM, Weichselbaum RR, Salama JK A rationale for the targeted treatment of oligometastases with radiotherapy. J Surg Oncol 2008; 98: 202–6. doi: 10.1002/jso.21102 18618604

[4] Bignardi M, Navarria P, Mancosu P, Cozzi L, Fogliata A, Tozzi A, et al Clinical outcome of hypofractionated stereotactic radiotherapy for abdominal lymph node metastases. Int J Radiat Oncol Biol Phys 2011; 81: 831–8. doi: 10.1016/j.ijrobp.2010.05.032 20800375

[5] Kim MS, Yoo SY, Cho CK, Yoo HJ, Yang KM, Kang JK, et al Stereotactic body radiotherapy for isolated para-aortic lymph node recurrence after curative resection in gastric cancer. J Korean Med Sci 2009; 24: 488–92. doi: 10.3346/jkms.2009.24.3.488 19543514 PMC2698197

[6] Benedict SH, Yenice KM, Followill D, Galvin JM, Hinson W, Kavanagh B, et al Stereotactic body radiation therapy: the report of AAPM task group 101. Med Phys 2010; 37: 4078–101. doi: 10.1118/1.3438081 20879569

[7] Ahmed KA, Torres-Roca JF Stereotactic body radiotherapy in the management of oligometastatic disease. Cancer Control 2016; 23: 21–9. doi: 10.1177/107327481602300105 27009453

[8] Almaghrabi MY, Supiot S, Paris F, Mahé MA, Rio E Stereotactic body radiation therapy for abdominal oligometastases: a biological and clinical review. Radiat Oncol 2012; 7: 126–36. doi: 10.1186/1748-717X-7-126 22852764 PMC3485144

[9] Niibe Y, Kenjo M, Kazumoto T, Michimoto K, Takayama M, Yamauchi C, et al Multi-institutional study of radiation therapy for isolated para-aortic lymph node recurrence in uterine cervical carcinoma: 84 subjects of a population of more than 5,000. Int J Radiat Oncol Biol Phys 2006; 66: 1366–9. doi: 10.1016/j.ijrobp.2006.07.1384 17126206

[10] Kim JS, Kim JS, Kim SY, Kim K, Cho MJ Hyperfractionated radiotherapy with concurrent chemotherapy for para-aortic lymph node recurrence in carcinoma of the cervix. Int J Radiat Oncol Biol Phys 2003; 55: 1247–53. doi: 10.1016/S0360-3016(02)04401-2 12654434

[11] Choi CW, Cho CK, Yoo SY, Kim MS, Yang KM, Yoo HJ, et al Image-guided stereotactic body radiation therapy in patients with isolated para-aortic lymph node metastases from uterine cervical and corpus cancer. Int J Radiat Oncol Biol Phys 2009; 74: 147–53. doi: 10.1016/j.ijrobp.2008.07.020 18990511

[12] Bignardi M, Navarria P, Mancosu P, Cozzi L, Fogliata A, Tozzi A, et al Clinical outcome of hypofractionated stereotactic radiotherapy for abdominal lymph node metastases. Int J Radiat Oncol Biol Phys 2011; 81: 831–8. doi: 10.1016/j.ijrobp.2010.05.032 20800375

[13] Sologuren I, Gallego CR, Lara PC Immune effects of high dose radiation treatment: implications of ionizing radiation on the development of bystander and abscopal effects. Transl Cancer Res 2014; 3: 18–31.

[14] Corbin KS, Hellman S, Weichselbaum RR Extracranial oligometastases: a subset of metastases curable with stereotactic radiotherapy. J Clin Oncol 2013; 31: 1384–90. doi: 10.1200/JCO.2012.45.9651 23460715

